# Computational framework for combining multiple swept-sources for high-resolution in-vivo optical coherence tomography

**DOI:** 10.1364/BOE.581000

**Published:** 2026-01-09

**Authors:** Sarvesh Thakur, Pepijn Klooster, Baris Bargu, Dierck Hillmann

**Affiliations:** Department of Physics, Vrije Universiteit Amsterdam, De Boelelaan 1105, 1081 HV Amsterdam, Netherlands

## Abstract

Axial resolution in swept-source-based Fourier-domain optical coherence tomography (FD-OCT) is limited by the sweep range of the source. However, broad swept-sources are not readily available, and passively combining two laser sources is not straightforward. In this paper, we develop a framework to overcome this limit by computationally combining independently sweeping sources to increase the bandwidth and subsequently the axial resolution of full-field Fourier-domain optical coherence tomography (FF-FD-OCT) systems. To this end, we demonstrate a dual-laser full-field FD-OCT system that uses two lasers, which sweep sequentially and are stitched phase-correctly in post-processing to obtain high-bandwidth spectra. After combining, we achieve an effective bandwidth of 145 nm at a central wavelength of 878 nm. The system has a high axial resolution of 3.1 *μ*m and can operate at an A-scan rate of 50 MHz. Our method requires a one-time calibration measurement to determine the non-linear sweeps from the lasers and the wavelength overlap, as well as a volume-by-volume phase matching procedure to compensate for sample motion. We demonstrate this for *ex-vivo* phantoms as well as *in-vivo* retinal data. Overall, the framework allows for extension to multiple lasers to further improve the axial resolution.

## 1. Introduction

Full-field Fourier-domain optical coherence tomography (FF-FD-OCT) enables fast and non-invasive imaging of biological tissues such as the *in-vivo* human retina [1–3]. Unlike conventional FD-OCT which uses confocal scanning to image the sample, FF-FD-OCT uses a high-speed area camera to acquire phase stable interferograms from all lateral points in parallel. The ability to recover the phase-correct wave-fields makes this technique particularly suitable for post-processing using computational imaging techniques such as digital refocusing [4] and aberration correction without having hardware-based adaptive optics [2]. Although computational adaptive optics (CAO) was first demonstrated in scanning OCT [5], scanning systems lack intrinsic lateral phase stability, and it is therefore either restricted to a single enface layer or requires sample motion tracking [6], which may not be possible when motion-induced gaps in data occur. State-of-the-art FF-FD-OCT systems are capable of reaching effective A-scan rates higher than 20MHz , making them an order of magnitude faster than scanning OCT systems [2,7–9] and can thereby inherently provide the phase stability needed for CAO.

One important application of FF-FD-OCT is in the emerging field of optoretinography (ORG) where functional signals from the retina are analyzed [10,11]. FF-FD-OCT can detect small changes in optical path lengths between different layers such as the photoreceptor outer segments [12]. Furthermore, FF-FD-OCT has also been used to study retinal biomechanics, including pulse wave propagation and retinal blood flow [13,14]. At present, most FF-FD-OCT systems designed for retinal imaging have a bandwidth of about 75nm around a central wavelength of ∼840nm which corresponds to an axial resolution down to 5μm in air [9,12,15]. This imposes a limitation in differentiating functional signals from more intricate layers in the retina, such as distinguishing the functional signals from cones and rod outer-segments tips in certain regions [12] or investigating the neuronal functions of the sub-layers of the inner-plexiform layer [10].

The lateral and axial resolution in OCT are decoupled. The lateral resolution depends on the imaging optics which, for retinal imaging, is restricted by the pupil of the eye after correcting for the aberrations. On the other hand axial resolution depends on the bandwidth of the source. However, the semiconductor optical amplifiers (SOAs) that are used inside the lasers have a limited bandwidth in the near-infrared region. For spectral domain scanning OCT systems, broader sources are available and can be achieved either by coupling multiple superluminescent diodes (SLDs) [16], by using a pulsed laser [17], or by using a supercontinuum source [18]. For scanning swept-source OCT systems, combining SOAs to increase resolution has not been successful so far, as the laser sweeps in 100s or even 1000s of kHz range have to be combined phase-correctly to achieve full resolution improvement. However, in FF-FD-OCT the lasers sweep at relatively slow speeds ( <200Hz ) where the sweeps stay sufficiently reproducible.

In this paper, we develop a framework to computationally combine independently sweeping sources to increase bandwidth of a FF-FD-OCT system. This allows us to acquire spectral raw data from sequentially triggered sources and then phase-correctly stitch the raw data in post-processing by matching phase and wavelengths. It results in a single high-bandwidth spectral raw dataset. The framework requires a reproducible trigger mechanism and then allows calibrating each laser to linearize the laser sweeps and afterwards determine the overlap for lasers with a common sweep range. It can further compensate the spectral phase changes due to sample motion between the sweeps, so we can stitch the spectra without any phase jumps.

To demonstrate our framework, we constructed a Dual-laser FF-FD-OCT system that uses two synchronized sweeping laser sources: Laser-840, which sweeps from 805nm to 882nm and Laser-950, which sweeps from 879nm to 950nm . Combining these, we achieve an effective axial resolution of 3.1μm in air, corresponding to a bandwidth of 145nm centered at 878nm . The resulting axial resolution of 3.1μm is a significant improvement compared to Laser-840 alone ( 5.5μm ) or Laser-950 alone ( 7.2μm ). To image samples, the data is acquired at the same laser settings as the calibration and stitched in post-processing to get the high-resolution reconstruction. We demonstrate the resolution improvement in both, calibration targets as well as *in-vivo* human retina.

## 2. Methods

### 2.1. Characteristics of signals acquired in FF-FD-OCT

In FF-FD-OCT, the camera records the interferograms I(t) at a fixed frame-rate while the laser sweeps over its bandwidth. In general, the laser sweep is non-linear in time and is given by k(t) . For such a setup, the OCT signal for a generic sample with scattering potential η(z) , after subtracting DC and auto-correlation terms is given by

(1) $$I(t)=S(k(t))\int_{-\infty }^{\infty }\eta (z)\;\cos[2k(t)z+\phi (k(t))]\;dz\text{.}$$

Here, k=2π/λ is the wavenumber, z is the depth, S(k) is the spectral shape of the source, and k(t) represents the non-linear sweep of the laser that we refer to as chirp. In addition a time varying phase factor ϕ(k(t)) is introduced by a combination of dispersion mismatch and bulk axial motion of the sample. Bulk axial motion in FF-FD-OCT is significant as the laser sweep speeds are slower compared to point scanning swept-source OCT (SS-OCT) systems.

The non-linear sweep k(t) leads to the acquisition of a chirped signal which causes a depth-dependent broadening of the axial point spread function (PSF) in the reconstruction. The phase term ϕ(k(t)) on the other hand causes a uniform broadening of the PSF that is independent across depths. As the sample motion contributes to ϕ(k(t)) , it cannot be calibrated before hand. However, this uniform blurring is an important property that allows us to optimize the sample phase by using a sharpness metric, comparable to determining motion and dispersion or aberrations [2]. To stitch acquired OCT spectra from independent swept-sources both k(t) and ϕ(t) need to be corrected for, which is further discussed in sections 2.4 and 2.5.

### 2.2. Axial resolution

The acquired OCT spectra are band-limited in wavenumber as the laser has a limited sweep range. Axial resolution in Fourier-domain OCT (FD-OCT) depends upon the bandwidth and the spectral shape of the source. For a tunable laser with a rectangular spectral output, the axial resolution as the full-width at half maximum (FWHM) of the point-spread function is given by

$$\Delta z_{\mathrm{FWHM}}=\frac{1.21\pi }{\Delta k_{0}},$$

where $\Delta k_{0}$ is the full sweep-range/bandwidth in wavenumbers. The FWHM scales inversely with bandwidth. Therefore, increasing the bandwidth of the source results in higher axial resolution.

### 2.3. Setup and data acquisition

**Fig. 1.**
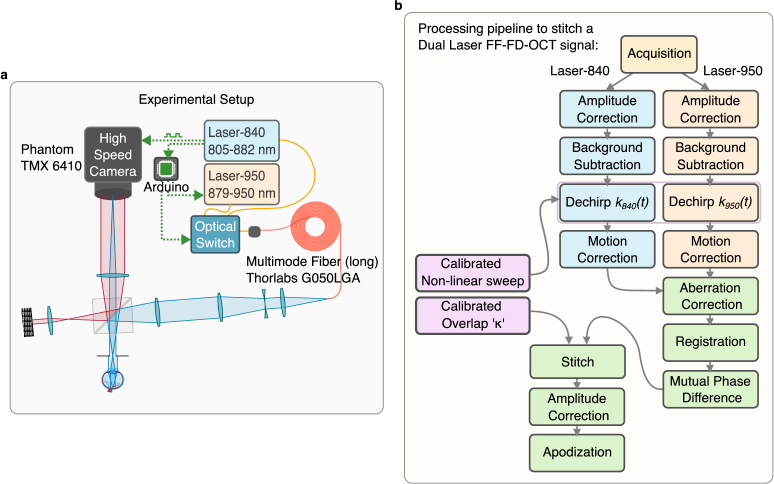
**a.** Schematic of the Dual-laser FF-FD-OCT system. **b.** Shows the entire processing pipeline from data acquisition to the final stitched high-bandwidth OCT spectra

The Dual-laser FF-FD-OCT shown in Fig. 1 uses two slightly overlapping sources. Laser-840 (SLDSources.com Broadsweeper BS-840-1-OEM) can sweep from 805−882nm and Laser-950 (Superlum Broadsweeper BS-930-1-HP) can sweep from 879−1050nm , but it was restricted to sweep from 879−950nm considering the reduced sensitivity of the camera and the reduced transmission through the vitreous body at longer wavelengths [19–21]. Each laser is coupled into an optical switch (Thorlabs OSW22-780E) that can choose between the two with a delay of approximately 1ms . Light from the switch is passed through a long multimode fiber ( 300m , Thorlabs FG050LGA) to reduce spatial coherence [15,22]. Further, the light is sent into a Michelson interferometer. As the lasers sweep, the high-speed camera (Phantom TMX 6410) records interference between the reference and the back-scattered light from the sample at 65,000 fps at up to 1024×800 pixels per frame. The setup can image at an effective A-Scan rate of 100MHz with a single laser and at 50MHz with Dual-laser.

The triggering mechanism has to be precise enough so that the same calibration measurements can be used for all measurements. For our setup, this works best when Laser-840 is the controller which triggers the camera and an Arduino Mega 2560 board that further triggers the optical switch and Laser-950. This is due to Laser-840 having a jitter of 10μs when externally triggered which is not present if the laser is free-running. On the Arduino side, timer 0 has to be disabled to prevent it from causing unintentional interrupts that cause additional jitter. The Arduino is further programmed such that the trigger signal from the laser starts a custom interrupt on the Arduino that first sends a signal to the optical switch and then after about 1ms to Laser-950. With this setup, the triggering is stable enough to about $1/16^{\text{th}}$ of a camera frame. The sweep speeds of the lasers are not required to be matched during acquisition, however their sweep-rate was chosen close to each other, so their sampling of the spectrum roughly matches. With these settings, Laser-840 sweeps for about 515 camera frames, followed by a gap of about 74 frames, and then Laser-950 sweeps for about 478 frames. Since the camera can be up to one frame earlier or later with respect to the first laser, it is important that both lasers sweep in the same sweep direction. This ensures that the overlap is constant for all measurements.

Human retinal measurements were performed on a healthy male volunteer aged 41. The pupil was not dilated. The research received a Non-WMO declaration from the Medical Ethics Review Committee (METC) of the Amsterdam UMC. The lateral resolution for NA of 0.1 was 5.3μm . The power at the corneal plane was 2.4mW which was well below the maximum permissible exposure (MPE).

#### 2.3.1. Amplitude/ASE background correction

In Eq. (1), it is desirable to have the spectral shape S(k(t)) close to a perfect rectangle to achieve the highest possible FWHM resolution. However, in practice the intensity emitted by the laser varies slightly over the sweep. Furthermore, the lasers emit a significant amount of incoherent amplified spontaneous emission (ASE) background towards the sweep ends. Incoherent background causes a loss in interference contrast, that could falsely be interpreted as an intensity modulation. Particularly when combining multiple lasers, their amplitudes have to be matched at the overlay to prevent strong and sudden changes in modulation contrast. Therefore, it becomes essential to isolate the effect of ASE from the OCT signal to extract the correct calibration curves and the overlap value. Apart from the ASE, other optical components such as attenuators and neutral density filters may have a wavelength dependent response which can also influence interference contrast and thus amplitude.

To correct for this, we performed an amplitude correction. It is carried out differently for scattering and reflecting samples. For samples with sufficiently distributed scattering potential over depth, like tissue we perform the amplitude correction as follows. If $I_{m}(x,y,k)$ is the raw signal from the $m^{\text{th}}$ source, then the normalized sum of the absolute values of each A-line gives an estimate of the envelope $\Gamma_{m}$ :

(2) $$\Gamma_{m}(k)=\sum_{x,y}|I_{m}(x,y,k)|$$

Normalizing $\Gamma_{m}$ gives us the interference contrast $\alpha_{m}$

(3) $$\alpha_{m}(k)=\frac{\Gamma_{m}(k)}{\Gamma_{m,\text{max}}}\text{,}$$

where $\Gamma_{m,\text{max}}$ is the maximum value of $\Gamma_{m}$ along k . To correct for the loss in contrast, we divide the raw data $I_{m}(x,y,k)$ by $\alpha_{m}(k)$ for each k after subtracting DC.

For samples with localized scattering potential like mirrors or glass cover-slips, we first Hilbert transform $I_{m}(x,y,k)$ to get the complex signal ${\tilde{I}}_{\text{m}}(x,y,k)$ . Then we filter ${\tilde{I}}_{\text{m}}(x,y,k)$ to isolate the reflection from a single surface for calculating $\Gamma_{m}$ . This is needed to remove the modulation caused by the interference of the two or more reflecting depths on the magnitude of the OCT spectrum. If multiple measurements are available, we average the resulting signal over all measurements before correction. This amplitude correction is done for all measurements, including the calibration.

### 2.4. Calibration

To coherently stitch the spectra from m sources, we need to obtain the same equidistant k -spacing along the wavenumber axis for all lasers. After that, we must algorithmically determine the exact amount of overlap in pixels for each pair of lasers having an overlapping bandwidth. A one-time calibration step is necessary to find the overlap, and to correct the non-linear k -spacing caused by the chirp k(t) .

#### 2.4.1. Chirp determination

To determine k(t) computationally, OCT signals from a single reflecting surface can be used, such as a mirror at depth $z_{0}$ . The scattering potential for a mirror is given by $\eta (z)=A_{0}\delta (z-z_{0})$ . Then, for the $m^{\text{th}}$ source, Eq. (1) simplifies to

(4) $$I_{m}(t)=S_{m}(k_{m}(t))\;\cos[2k_{m}(t)z_{0}+\phi (k_{m}(t))]\text{.}$$

As long as $z_{0}$ is sufficiently below the Nyquist limit, we can get the analytical signals for Eq. (4) by adding its Hilbert transform as imaginary part. This gives

(5) $${\tilde{I}}_{m}(t)=S_{m}(k_{m}(t))\;e^{i(2k_{m}(t)z_{0}+\phi (k_{m}(t)))}\text{.}$$

The argument of Eq. (5) has two unknowns, $k_{m}(t)$ and $\phi_{m}(k_{m}(t))$ . In the absence of motion and dispersion, the phase term from Eq. (5) drops out and the OCT signal from a single depth $z_{0}$ becomes:

(6) $${\tilde{I}}_{m}(t)=S_{m}(k_{m}(t))\;e^{i2k_{m}(t)z_{0}}$$

The argument of Eq. (6) is then unwrapped and used to extract $k_{m}(t)$ and the spectrum is resampled with the chirp curve using a gridding algorithm as used in non-equispaced fast Fourier transforms (NFFT, [23–25]) along the time axis to get a signal with equidistant k -spacing, ${\tilde{I}}_{m}(k)$ . We also ensure that all resampled spectra have the same pixel size δk after the resampling. Before resampling the data, both lasers have slightly different depth ranges as the laser’s sweep rates differ. Once the sampling density for both lasers is matched to δk , a depth $z_{0}$ generates a signal of constant frequency. The resampling step acts like a computational k -clock which not only linearizes the signals but also matches their sweep rate. After the resampling, if we zero pad the data from one laser to match the size of the other, we see the same structures at the same depth for both lasers. Essentially, all calibration signals after regridding become

(7) $${\tilde{I}}_{m}(k)=S_{m}(k)\;e^{i2kz_{0}}\;\text{with }k\in [k_{m}^{\max},k_{m}^{\min}]\text{,}$$

with a single $z_{0}$ .

#### 2.4.2. Overlap determination

Sources m and m+1 have a common wavenumber region which we will refer to as their overlap. For convenience, we calculate the overlap directly in pixels, therefore k -linearization is done before determining the overlap. After k -linearization, with each pixel representing a fixed wavenumber range δk , the signals from source m and m+1 get lengths $N_{m}$ and $N_{m+1}$ , respectively.

**Fig. 2.**
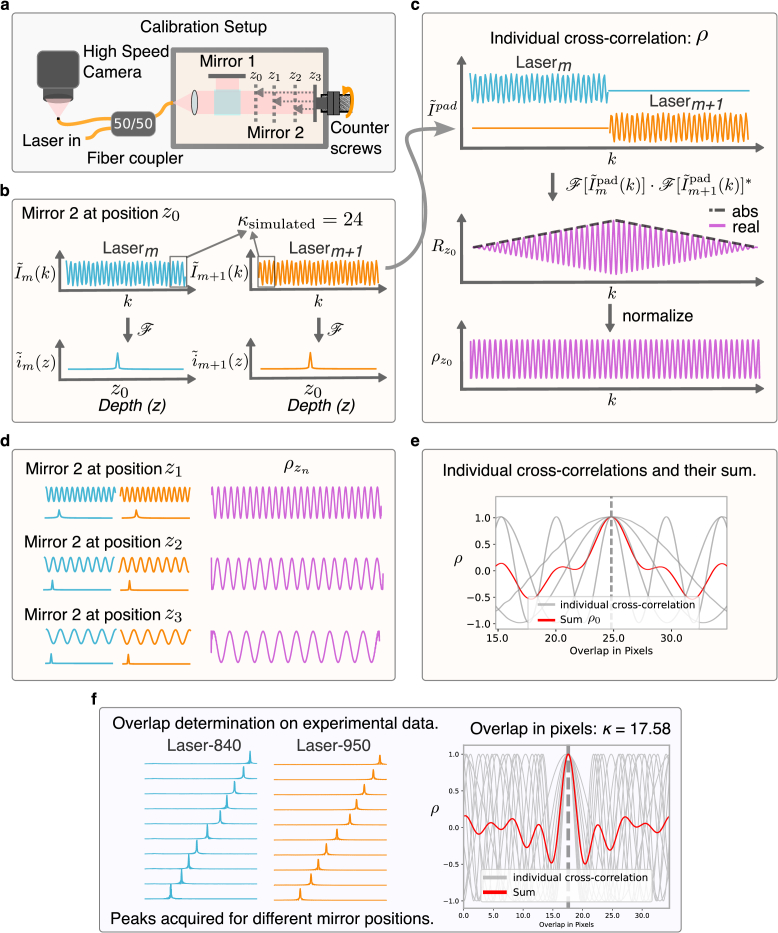
Overlap determination procedure **a.** Setup for acquiring calibration data. Output from a stable Michelson interferometer is recorded at different depths. **b.** Simulated k -linearized OCT signals ${\tilde{I}}_{m}$ and ${\tilde{I}}_{m+1}$ with overlap κ equivalent to 24 pixels for mirror position $z_{0}$ and its corresponding reconstruction, i.e., a fast Fourier transform along the k -axis. **c.** Calculation of normalized cross-correlation $\rho_{z_{0}}$ between the padded signals ${\tilde{I}}_{m}^{\text{pad}}$ and ${\tilde{I}}_{m+1}^{\text{pad}}$ . **d.** The normalized cross-correlations for all the other positions $z_{1}-z_{3}$ are calculated similarly. **e.** The individual cross-correlations add up constructively at the simulated overlap κ=24 pixels. **f.** Method applied on experimental data. Peaks from 28 different depths were acquired from a stable Michelson interferometer. Overlap determined to be 17.58 pixels.

A phase stable OCT signal originating from multiple reflecting layers that are distributed over uncorrelated non-harmonic depths, creates a unique fingerprint in the overlap region. Therefore, we can determine the overlap between two adjacent sources m and m+1 by summing the cross-correlation of n padded and k -linearized OCT signals acquired from the from different depths $z_{n}$ . At first, the k -linearized signals from both lasers ${\tilde{I}}_{m}(k)$ and ${\tilde{I}}_{m+1}(k)$ are padded to the same length $N_{m}+N_{m+1}$ as shown in Fig. 2(c):

(8) $${\tilde{I}}_{m}^{\text{pad}}(k)=\left\{ \begin{array}{ll} {\tilde{I}}_{m}(k), & k\in [0,\;N_{m}]\\ 0 & \text{otherwise}, \end{array}\right.\;{\tilde{I}}_{m+1}^{\text{pad}}(k)=\left\{ \begin{array}{ll} {\tilde{I}}_{m+1}(k), & k\in [N_{m},\;N_{m}+N_{m+1}]\\ 0 & \text{otherwise}. \end{array}\right.$$

Next, as illustrated in Fig. 2(c,d), we compute the individual cross-correlation R between ${\tilde{I}}_{m}^{\text{pad}}(k)$ and ${\tilde{I}}_{m+1}^{\text{pad}}(k)$ obtained from a single mirror position $z_{n}$ by using the convolution theorem as follows

(9) $$R_{z_{n}}={\tilde{I}}_{m}^{\text{pad}}(k)⋆{\tilde{I}}_{m+1}^{\text{pad}}(k)=F^{-1}\left[F[{\tilde{I}}_{m}^{\text{pad}}(k)]\cdot F[{\tilde{I}}_{m+1}^{\text{pad}}(k){]}^{*}\right]$$

The padding before calculating the cross-correlation ensures that the effectively calculated circular cross-correlation is prevented. In Eq. (9), before taking the inverse Fourier transform, the quantity $F[{\tilde{I}}_{m}^{\text{pad}}(k)]\cdot F[{\tilde{I}}_{m+1}^{\text{pad}}(k){]}^{*}$ is further zero-padded 99 times to get a resolution of 0.01 pixels in the cross-correlation. Now, we assume that the padded spectrum for the first laser is given by

(10) $${\tilde{I}}_{m}^{\text{pad}}(k)=S_{m}(k)\;e^{i2kz_{n}},$$

where $S_{m}(k)$ is given by a combination of the light source and the padding (where it is =0 ) and will approximately resemble a rectangular-function. Since the acquisition was dispersion-free, $S_{m}(k)$ is purely real and $S_{m}(k)\geq 0$ . Similarly, the padded spectrum from the second laser, will be given by

(11) $${\tilde{I}}_{m+1}^{\text{pad}}(k)=S_{m+1}(k)\;e^{i2(k+\kappa )z_{n}},$$

where κ is the unknown number of pixels in which the spectrum is shifted compared to the first laser. It thus corresponds to the overlap. The other parameters correspond to the situation in the first laser, notably k and $z_{n}$ are identical.

With this, Eq. (9) becomes

(12) $$R_{z_{n}}=F^{-1}\left[F[S_{m}(k)\;e^{i2kz_{n}}]\cdot F[S_{m+1}(k)\;e^{i2(k+\kappa )z_{n}}{]}^{*}\right]\text{.}$$

We can rewrite this as

(13) $$R_{z_{n}}=F^{-1}\left[F[S_{m}(k)\;e^{i2kz_{n}}]\cdot F[S_{m+1}(k)\;e^{i2kz_{n}}{]}^{*}\right]e^{-i2\kappa z_{n}}\text{.}$$

A linear phase ramp $e^{i2kz_{n}}$ shifts the transformed output by $2z_{n}$ (Fourier shift theorem), which gives

(14) $$R_{z_{n}}=F^{-1}\left[{\tilde{s}}_{m}(z-2z_{n})\cdot {\tilde{s}}_{m+1}^{*}(z-2z_{n})e^{i2\kappa z_{n}}\right]\text{,}$$

where ${\tilde{s}}_{m}(z)=F[S_{m}(k)]$ . This can be rewritten as

(15) $$R_{z_{n}}(k)=(S_{m}⋆S_{m+1})\exp(i2z_{n}(k-\kappa ))\text{.}$$

Since $S_{m}$ and $S_{m+1}$ are real and non-negative, so is $S_{m}⋆S_{m+1}$ . Consequently, $R_{z_{n}}$ has two components, $S_{m}⋆S_{m+1}$ that depends on the convolution of spectral envelopes of the two sources and a carrier frequency $e^{2z_{n}i(k-\kappa )}$ . For rectangular spectral shapes the convolution results in a triangular shape for $S_{m}⋆S_{m+1}$ as seen in Fig. 2(c), however, in practice this shape can vary slightly. Equation (15) can in either case be normalized by dividing with its absolute value. Assuming that there is no additional phase component we can also take the real part, giving

(16) ρzn(k)=Re(Rzn)(k)|Rzn(k)|=cos⁡(2(k−κ)zn).

We call Eq. (16) the individual normalized cross-correlation between the two laser spectra acquired from a single depth $z_{n}$ . The normalized cross-correlation from a single depth is periodic and does not reveal information about the overlap. To find the overlap, OCT signals from several such peaks are recorded (Fig. 2(b,d)) and the resulting normalized cross-correlations $\rho_{z_{n}}$ are added. The resulting sum is the total cross-correlation from N different depths,

(17) $$\rho_{0}(k)=\sum_{n=0}^{N-1}\rho_{z_{n}}(k)=\sum_{n=0}^{N-1}\cos(2(k-\kappa )z_{n})\text{.}$$

It adds up constructively at the point of overlap (Fig. 2(e)) κ , assuming that $z_{n}$ are not multiples of each other. The overlap $\kappa_{0}$ can thus be predicted by

(18) $$\kappa_{0}=\underset{k}{\arg\max}\;\rho_{0}(k)\approx \kappa .$$

#### 2.4.3. Calibration setup and acquisition

The setup for calibration is illustrated in Fig. 2(a). The light from the two sources is coupled into a stable Michelson interferometer through a 50:50 coupler and the output from the interferometer is projected through the same coupler and a bare single-mode fiber onto the camera. The stable Michelson interferometer is milled from a solid block of aluminium, which has a dispersion-free design and two counter screws to lock mirrors at a certain path length difference to eliminate motion artifacts. The triggering mechanism and the laser parameters are kept the same as intended for imaging actual samples. The mirror is locked in at a fixed depth and the OCT signal from both lasers is recorded sequentially. This is repeated for 28 different depths which are the values for $z_{n}$ . After that, for each laser, all A-lines in a single volume are averaged. A few camera images near the laser sweep start and stop points are discarded due to the uncertainty of the laser sweep start, and in order to maintain the same amount of overlap if the camera misses a trigger by a single frame. This can happen as the camera is set to run freely at a constant frame rate which is not synchronized with the laser trigger. Figure 2(f) shows the overlap determination for experimental data measured from the 28 different depths. Later analysis showed that approximately 6 different depths would have been enough to determine the overlap for our lasers and imaging parameters. However, the larger dataset for the one-time calibration provides a robust estimate and does not adversely affect the results. The calibrated curves and the overlap values stay constant as long as the sweep speeds stay the same.

While theoretically, both chirp calibration and overlap determination could be done by a measurement taken from a single calibration target having multiple reflecting layers, in practice this did not work: The cross-correlation from the resulting signal cannot be normalized since the individual signals will interfere; alternatively, windowing peaks to isolate them results in a loss of spectral resolution. Both cases cause bad edge behavior which does not work well with overlap determination as the overlap region lies towards the sweep ends. Further, in a full-field system, there are challenges with isolating stray reflections and getting rid of sample motion and dispersion without altering the phase. Therefore, we choose to acquire data from each mirror position separately using the stable Michelson interferometer to determine the overlap. The use of individual mirror positions allows for the normalized and modified cross-correlation to be used and that turned out to be crucial for a robust determination of the small overlap of a few pixels.

Once the k(t) and the overlap have been determined, the calibration signals can be stitched by moving one of the laser spectra to the overlap position using the Fourier-shift theorem. A smooth curve is used in the overlap region to remove any jumps in amplitude. For samples other than calibration targets, phase errors have to be removed before stitching which is discussed in section 2.5.

### 2.5. Reconstruction of high-resolution OCT images

Since the calibration already measures k(t) and the overlap κ , only the phase errors arising from sample motion need to be corrected before stitching actual samples.

Figure 1(b) gives an outline of the processing pipeline for a single volume. Measurements are acquired at the exact same triggering and sweep parameters as for the calibration. Typically, several volumes are recorded for a single sample to increase signal-to-noise ratio (SNR). Every volume contains two sweeps from each laser with a fixed time gap.

At first, the acquired spectra are split by laser channel and background subtracted [26], followed by contrast correction to compensate ASE. After that, both the spectra are k -linearized by resampling to have an equidistant k -spacing per pixel. The OCT signal of a sample with scattering potential η(z) and from source m is given by

(19) $${\tilde{I}}_{m}(k)=\int_{-\infty }^{\infty }\eta (z)e^{i(2kz+\phi_{m}(k))}\;dz\text{.}$$

During acquisition, the sample moves causing phase errors in the measured OCT signal. Axial bulk motion from the sample and dispersion mismatch in the interferometer introduce a phase component ϕ(k) in the OCT signal. ϕ(k) can be expressed as a Taylor expansion around a central wavelength $k_{0}$ ,

(20) $$\phi (k)=\phi (k_{0}+\Delta k)=\phi (k_{0})+{\frac{\partial \phi (k)}{\partial k}|}_{k=k_{0}}\;\Delta k+O\left(\Delta k^{2}\right).$$

In Eq. (20), the higher order terms $O\left(\Delta k^{2}\right)$ represent a combined effect of in-volume sample motion and dispersion mismatch. This is corrected for each volume and for each laser separately using a motion correction approach described in [27]. After motion correction, we are left with the linear term $\phi^{\prime }(k_{0})\;\Delta k$ and a phase offset $\phi (k_{0})$ . The linear phase ramp $\phi^{\prime }(k_{0})\;\Delta k$ gives the axial displacement between two volumes. The linear phase ramp is removed by registering all volumes to one another. From a total of N volumes acquired from M laser sources, we create a list of N×M volumes and mutually register them to get rid of phase ramps. Finally, the signals from adjacent sources m and m+1 are only left with a mutual phase offset that needs to be matched. The signals can be represented as follows

(21) $${\tilde{I}}_{m}(k)=\int_{-\infty }^{\infty }\eta (z)e^{i(2kz+\phi_{0,m})}\;dz$$

(22) $$\text{and}\;{\tilde{I}}_{m+1}(k)=\int_{-\infty }^{\infty }\eta (z)e^{i(2(k+\kappa )z+\phi_{0,m+1})}\;dz\text{,}$$

where only a constant phase is left to be estimated. As κ is known, we use the Fourier shift theorem to shift back ${\tilde{I}}_{m+1}(k)$ by κ pixels giving us

(23) $${\tilde{I}}_{m+1}(k)=\int_{-\infty }^{\infty }\eta (z)e^{i(2kz+\phi_{0,m+1})}\;dz\text{.}$$

To stitch two volumes, the spectra have to be devoid of any phase jumps which originate from sample motion. Therefore, the constant phase offsets $\phi_{0,m}$ and $\phi_{0,m+1}$ must match. We assume that $\phi_{0,m}$ is the ground truth and perform a grid-search for $\phi_{0,m+1}$ . To find the phase offset, each registered volume is divided into lateral bins of $N_{x}$ x $N_{y}$ pixels giving local volumes ${\tilde{I}}_{m}^{\text{loc}}(x,y,k)$ and ${\tilde{I}}_{m+1}^{\text{loc}}(x,y,k)$ and then the phase for each of these local volumes is determined independently. For this, a $\phi_{\text{guess}}\in [0,2\pi )$ is applied to ${\tilde{I}}_{m+1}^{\text{loc}}(k)$ and the spectra is stitched as follows

(24) $${\tilde{I}}_{m,m+1}^{\text{loc}}(k)=\left\{ \begin{array}{ll} {\tilde{I}}_{m}^{\text{loc}}(k), & k\in [0,\;N_{m}-\kappa ),\\ {}[1-w(k)]{\tilde{I}}_{m}^{\text{loc}}(k)+w(k){\tilde{I}}_{m+1}^{\text{loc}}(k)\;e^{i\phi_{\text{guess}}}, & k\in [N_{m}-\kappa ,\;N_{m}],\\ {\tilde{I}}_{m+1}^{\text{loc}}(k)\;e^{i\phi_{\text{guess}}}, & k\in (N_{m},\;N_{m+1}-\kappa ]\text{,} \end{array}\right.$$

where ${\tilde{I}}_{m,m+1}^{\text{loc}}(k)$ is the stitched spectra for a local bin and w(k) is a weighting function to avoid intensity jumps and can, e.g., be a linear ramp

(25) $$w(k)=\frac{k-(N_{m}-\kappa )}{\kappa }\text{.}$$

From that, we then reconstruct the local OCT volume by

(26) $$U^{\text{loc}}(x,y,z)=F^{-1}[{\tilde{I}}_{m,m+1}^{\text{loc}}(k)]\text{.}$$

For this, we can evaluate a sharpness metric that tells us how well side-lobes are suppressed. A bad phase match, will cause significant side-lobes whereas a correct phase matching will minimize them. Thus, we use the sharpness metric [28] as follows

(27) $$S=\sum_{x,y,z}|U^{\text{loc}}(x,y,z){|}^{\gamma }\text{,}$$

where γ=1.4 gave good results. By minimizing this, we find the optimum value of $\phi_{\text{guess}}$ for each local bin [28].

For the fields of view and motion we encountered, dividing into bins of $N_{x}=25$ and $N_{y}=25$ pixels, corresponding to 150 μm×150 μm gave good results. In addition ${\tilde{I}}^{\text{loc}}(x,y,k)$ was zero-padded such that the total pixels in depth became 4× the original length. Zero-padding by at least a factor of 2× is recommended as the sharpness metric performs the best if sidelobes are visible. We obtain the stitched spectra ${\tilde{I}}_{\text{stitched}}(k)$ by applying the correct phase offsets across all lateral locations and stitching the spectra using Eq. (24) at the predetermined overlap value obtained from Eq. (18). Finally, for retinal images the spectra are amplitude corrected again using the method described in section 2.3.1 and apodized with a Tukey window with a ratio of 0.5 to reduce sidelobes.

Once the dataset is loaded into memory, the reconstruction of 50 volumes having 512×512 pixels laterally and cropped around the retina in depth takes about 19 minutes on a computer with 128 GB of RAM and NVIDIA RTX A5000 GPU. The total computation time is divided as follows, the k -linear reconstruction along with background subtraction takes 40% , followed by aberration correction which takes 2% , then the registration takes 18% and finally the phase search along with stitching takes 40% of the total time. For larger datasets, repeated transfers between GPU (25 GB) and CPU memory slow down the reconstruction. The larger field-of-view dataset with 1024 x 800 lateral pixel dimensions has a size of 80 GB after cropping around the retina and it takes 100 minutes to reconstruct due to memory limitations of the GPU.

## 3. Results

**Fig. 3.**
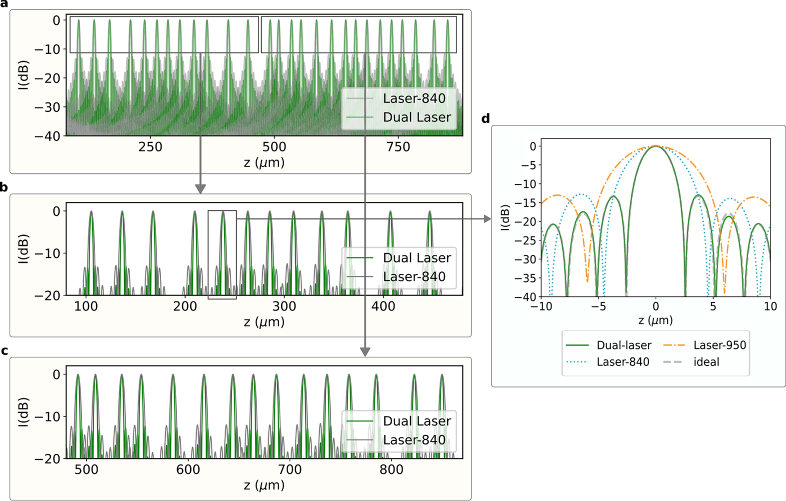
**a.** Comparison of zero-padded PSFs in air for Laser-840 and Dual-laser across the entire measurement depth and at different depths of the calibration data. **b** & **c.** Zoomed-in sections of a. **d.** Comparison of a single PSF for Laser-950, Laser-840, Dual-laser and the ideal bandlimited signal.

Figure 3 shows the results of validation of the calibration procedure on itself. Zero-padded PSFs of the peaks at different depths acquired by the stable Michelson are compared between the Dual-laser and Laser-840 signals. The Dual-laser PSF at all depths is consistently narrower while keeping sidelobes at the same threshold as its single laser counterpart. Figure 3(d) shows a single PSF for Dual-laser, Laser-840, Laser-950 and an ideal bandlimited signal. Laser-840 ( 805−880nm ) has a FWHM ( −6dB ) resolution of 5.4μm whereas Laser-950 ( 879−950nm ) has a resolution of 7.2μm . The Dual-laser signal has a higher resolution of 3.1μm which is equal to the expected theoretical resolution limit for the bandlimited rectangular spectrum in air.

**Fig. 4.**
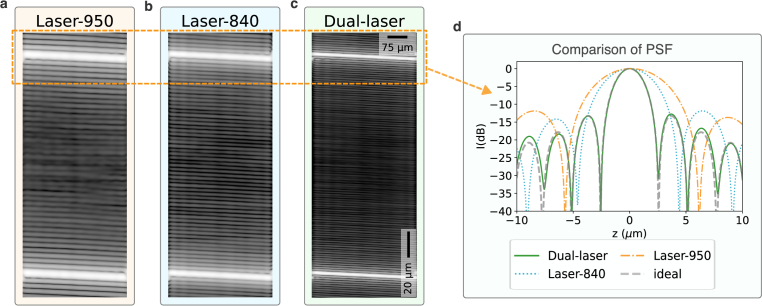
**a.-c.** Comparison of zero-padded B-Scans for a 170μm thick glass coverslip acquired with Laser-950 (a), Laser-840 (b), and Dual-laser (c). **d.** Extracted PSFs of the upper peak of the coverslip for Laser-950, Laser-840 and Dual-laser along with the ideal bandlimited signal for the combined sweep range

To validate the technique and the resulting PSF on samples imaged independent of the calibration data, it was tested on a coverslip target, giving two isolated reflecting layers. A Thorlabs precision coverglass with a known thickness of 170±5μm was used. Figure 4(a, b, and c) shows the B-scans of the coverslip from Laser-840, Laser-950 and Dual-laser, respectively. Figure 4(d) shows the comparison of PSFs from the upper reflecting surface of the coverslip. The FWHM of the PSF obtained from the Dual-laser reconstruction achieves the same 3.1μm , closely matching the ideal bandlimited signal. For both Figs. 3 and 4, the depth scaling was determined from the thickness of the coverslip which is known to be 170μm with a refractive index of 1.51 .

### 3.1. Effects of various errors while stitching the spectra

**Fig. 5.**
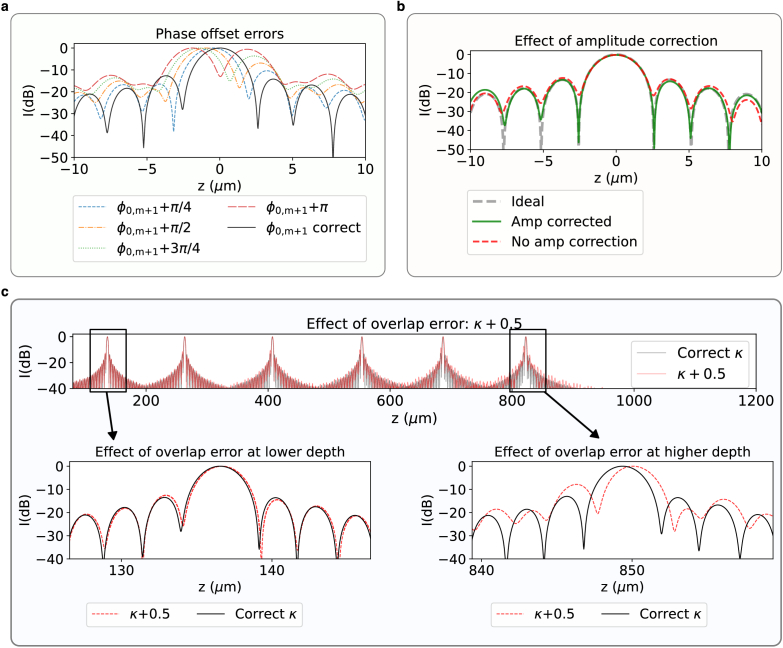
**a.** Effect of phase offset errors on calibration data. A phase offset was applied on source m+1 before stitching, in π/4 increments. **b.** Effect of amplitude variation on the axial PSF of the coverslip. **c.** Effect of stitching the sources at an overlap value off by 0.5 pixels.

Determining phase offset and overlap correctly, as well as amplitude correction are crucial for optimal results. Each stitching error degrades the axial PSF in a distinct way. An incorrect phase offset causes blurring of the axial PSF that is uniform across all depths. Figure 5(a) shows the effect of introducing phase offset errors from 0 to π in steps of π/4 on calibration data. A phase offset error of π/4 between the two lasers causes the sidelobes to rise on one side of the PSF by 3dB . This continues till the main lobe completely disappears and splits into two at an error of π .

Figure 5(b) shows the effect of amplitude correction on the coverslip. Amplitude variation in the spectra results in a convolution of the PSF, and having variations or even jumps in spectral intensities can cause increased sidelobes of the axial PSF. The uncorrected version has higher sidelobes which vanishes after flattening the spectral amplitude.

Unlike phase offset errors which cause PSF blurring across all depths, overlap errors result in degradation of the axial PSF which is depth dependent. Figure 5(b) shows the effect of stitching the calibration data at an overlap that is off by 0.5 pixels. At lower depths we do not see a significant effect for stitching with a incorrect overlap. At higher depths of 850μm the axial PSF a mistake of 0.5 pixel degrades the axial PSF significantly and increases the sidelobe on one side by approx. 5dB .

### 3.2. High-resolution imaging of in-vivo retina

**Fig. 6.**
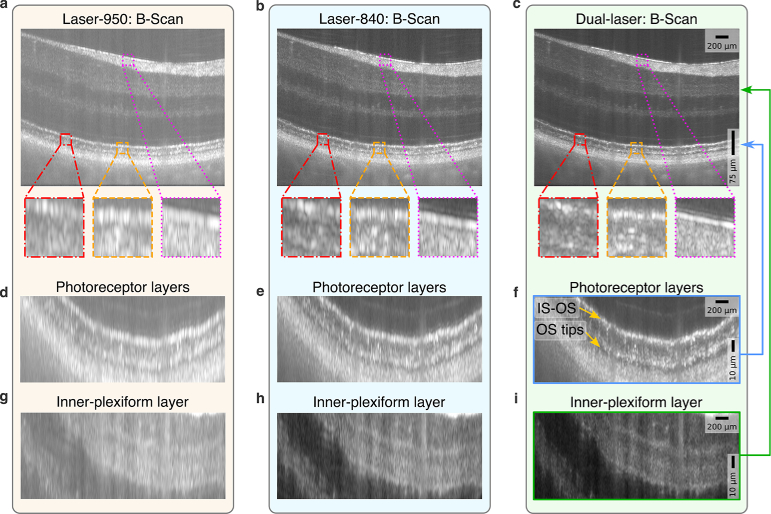
Resolution improvement observed in *in-vivo* retinal B-Scans. **a**–**c.** Comparison of an average of 50 volumes and 5 adjacent retinal B-Scans for Laser-950 (a), Laser-840 (b), and Dual-laser (c). Boxes below show zoomed in areas from the B-Scans with red and yellow boxes showing OS tips and IS-OS, whereas the magenta box shows the region near the NFL. **d**–**f.** Zoomed in region around the photoreceptor layers. **g**–**i.** Zoomed in region for an average of 50 volumes and 15 adjacent B-Scans around the inner plexiform layer (IPL). See also Visualization 1.

Finally, the technique was demonstrated for *in-vivo* retinal imaging. 50 volumes were acquired for a healthy living human retina for each laser which were stitched in post-processing to get high-resolution volumetric images, and the averaged volumes are displayed here. Figures 6(a), b, and c shows the comparison of retinal B-Scans for Laser-950, Laser-840, and Dual-laser reconstruction, respectively. The images shown are averaged from five adjacent B-Scans to reduce speckle noise. Further, the B-Scans are padded 3 times to better visualize side-lobes. Dual-laser images show better axial separation of layers and reduced speckle size. Below the B-Scans, red and yellow boxes compare the zoomed in region around the photoreceptor outer segment (OS) and the inner-segment outer-segment (IS-OS), whereas the magenta box shows the nerve fiber layer (NFL). Figures 6(d, e, and f) further show expanded view of the photoreceptor and surrounding layers, respectively, for clearer visualization. The photoreceptor IS-OS tips also show noticeably better axial separation in Dual-laser (Fig. 6(f)) compared to Laser-840 and Laser-950. Figures 6(g, h, and i) show averages of 15 adjacent B-Scans in the inner-plexiform layer (IPL). The Dual-laser images show an improvement in the resolution, reduced speckle size and higher contrast of sub-layers in the IPL.

**Fig. 7.**
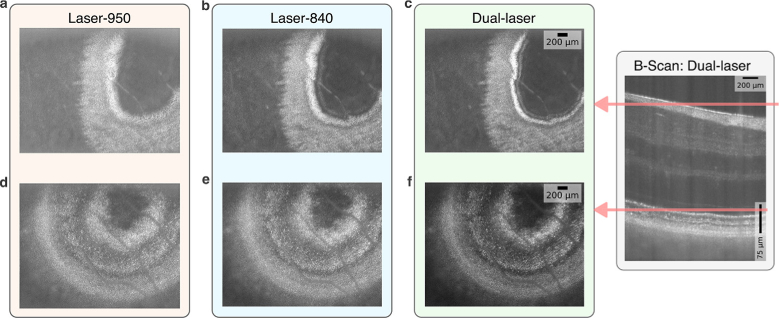
Comparison of enface layers of *in-vivo* retina for Laser-950, Laser-840 and Dual-laser. Higher axial resolution results in narrower cross-sections of layers laterally. **a**–**c.** Enface images for NFL layer as indicated by the red line on the B-Scan. **d**–**f.** Enface images of the cross-section of IS-OS layer. See also Visualization 1.

As a validation, enface images for NFL and IS-OS were also compared in Fig. 7 for single laser and Dual-laser reconstructions. The B-Scan on the right indicates the location of the enface layers in depth. Dual-laser reconstructions show thinner structures in the NFL (Fig. 7(a, b, and c)) and IS-OS layer (Fig. 7(d, e, and f)).

## 4. Discussion

We tested the proposed computational framework for combining multiple sources with a Dual-laser system comprising of Laser-840 ( 805−880nm ) and Laser-950 ( 879−950nm ). Despite having a relatively small overlap of about a nanometer in wavelength, we were able to successfully trigger the setup, calibrate the lasers, and phase-correctly stitch the spectra to increase the bandwidth. It took considerable effort to trigger the setup reliably to ensure that the calibration step can be independent of sample measurements. The modifications to the setup are simple, they include re-routing the lasers through an optical switch and, more importantly, re-configuring the triggering mechanism on the Arduino so that the sweeps are reproducible. The setup can be readily switched between single laser and Dual-laser imaging enabling the selection of faster imaging speed or higher axial resolution. At the present, the calibration has only been been tested to work at the exact same triggering parameters and sweep speeds. Axial resolution (in air) after combining the sources is 3.1μm which is a 1.7 times improvement over 5.5μm for Laser-840 alone and is in accordance with the expected theoretical value. For the calibration data, the resolution improved across the entire imaging depth while keeping the sidelobe suppression below −12dB which is close to the expected theoretical suppression of −13.26dB for a rectangular spectrum. Consistent improvement across the entire depth substantiates the accuracy of the k -linearization and overlap determination. The PSF from the cover-slip target shows that the images reconstructed from stitched spectra closely resemble those from a theoretically ideal signal with the combined bandwidth of the two lasers.

The framework is robust enough for *in-vivo* imaging of the human retina despite the effects of rapid eye motion which causes phase jumps between the two sources and across volumes. With two lasers the acquisition time for a single volume increases from 8ms to 17ms . This reduces the maximum effective A-scan rate for the system from about 100MHz to about 50MHz amplifying the effects of axial bulk motion originating from the sample. Motion correction, followed by registration and, finally, the local phase offset correction form a layered processing pipeline to remove phase artifacts. After stitching the spectra, the reconstructed images show a visible improvement in axial sharpness and have a smaller speckle size compared to single laser images. It is important to note that smaller speckle size is derived purely from the bandwidth and does not indicate how well the data from the two sources are combined. B-scans stitched with a phase jump in the spectra will also show smaller speckle size without the resolution improvement. Axially, Dual-laser images can resolve individual photoreceptor outer-segment tips in the inner segment/outer segment (IS/OS) junction as well as the outer segment (OS) tips (Fig. 6(f)). Resolution improvement can also be verified by comparing the thickness of different layers in *en-face* images such as the NFL or the IS-OS junction (Fig. 7). Highly reflecting layers such as the nerve fiber layer (NFL) show noticeable sidelobes in the Dual-laser reconstruction which could be improved in the future. The sub layers of the inner plexiform layer (IPL) are better resolved in the Dual-laser reconstruction (Fig. 6(i)). Apart from the improved resolution, weakly scattering layers like the IPL also show a higher contrast in the Dual-laser images. This can be attributed to having more sample light by virtue of having combined two sources.

For the stitching to be successful, we must accurately calibrate the non-linear chirp, find the overlap and finally the phase offset. Non-linear chirp determination is independent of the amount of overlap, although the chirp is sometimes less accurate towards the edges of the spectra. We do not expect a significant improvement in resulting image quality by having more or less overlap between the sources as long as the overlap and non-linear sweep have been calibrated correctly. However, a larger overlap could make the calibration part of the method more robust, and one may be able to work with fewer calibration measurements.

For the same acquisition speed, Dual-laser acquisition approximately doubles the amount of data acquired. Larger data size combined with additional processing steps increases the processing time for a dataset of 50 volumes cropped around the retina with lateral dimension 512×512 from 3min for single-laser FF-FD-OCT to 19min for Dual-laser FF-FD-OCT. Much of the increased processing time is due to limited GPU memory for handling large arrays. However, it should also be noted that the algorithms are not fully optimized at all steps, and these processing times are for quite significant data set sizes and correspond to 22 B-scans/s.

## 5. Conclusion

Previously, combining multiple laser sources to increase bandwidth was not easily possible in SS-OCT systems and was restricted to SD-OCT systems. Swept-source systems suffered from limited resolution due to the bandwidth limitations of SOAs used inside the lasers. In swept-source systems, combining lasers electronically or computationally to increase the bandwidth is a non-trivial task, due the varying triggering mechanisms, difference in characteristics of the lasers and sample motion. In this paper we have presented a computational framework to overcome the bandwidth limit of swept-sources in FF-FD-OCT by stitching the spectra from two or more independently sweeping lasers having an overlapping bandwidth. We achieved this by leveraging the low sweep speeds of the swept-source lasers in FF-FD-OCT systems which ensures sweep reproducibility and phase stability. However, in principle the framework can also work on scanning systems with a sufficiently stable swept-source and data acquisition, particularly since motion artifacts that alter phase hardly play a role there. We have shown that the framework can be applied to our Dual-laser setup to perform high-axial resolution OCT imaging of static samples as well as *in-vivo* imaging of the human retina. The resulting volumes have axial resolution equivalent to ideal spectra of the combined bandwidth and are phase stable.

FF-FD-OCT has already established itself to be suitable for non-invasive functional imaging of the human retina [10,11]. Our framework has the potential to improve upon that by being capable of extracting functional signals from finer sub-layers of the retina giving us more insights into the working of human visionary pathways. Increased resolution could be particularly useful in differentiating functional signals from rod and cone photoreceptor cells or studying the functional signals from the sub-layers of the IPL which is axially divided into five different strata having the neural connections that are participating in early signal processing such as responding to temporal stimuli [29]. Besides increasing the axial resolution, the Dual-laser volumes can also be axially averaged to decrease speckle noise. In principle, the framework should work for more than two lasers to further increase axial resolution until sample motion becomes too significant due to of longer acquisition times. The detector sensitivity and the scattering properties, e.g., water absorption, of the sample also play a limiting role in increasing the bandwidth further. In the future, we hope that our framework opens up new avenues for functional imaging of the retina by separating functional layers in the IPL, among others.

## Supplemental information

Visualization 1 This visualization shows a looping animation of retinal B-scans and enface images from Dual-laser and Laser-840. The red and yellow boxes show the zoomed in B-scans of IS-OS and OS-tips region. The regions surrounding photoreceptor segments and the i https://doi.org/10.6084/m9.figshare.30273388

## Data Availability

The underlying data and the demo code for the essential process are available from the authors upon reasonable request.
