# Supplementary figures and images for: Computational framework for combining multiple swept-sources for high-resolution in-vivo optical coherence tomography
Source: Biomed Opt Express. 2026 Jan 9;17(2):686–702. doi: 10.1364/BOE.581000 (PMC12904556; doi:10.1364/BOE.581000)

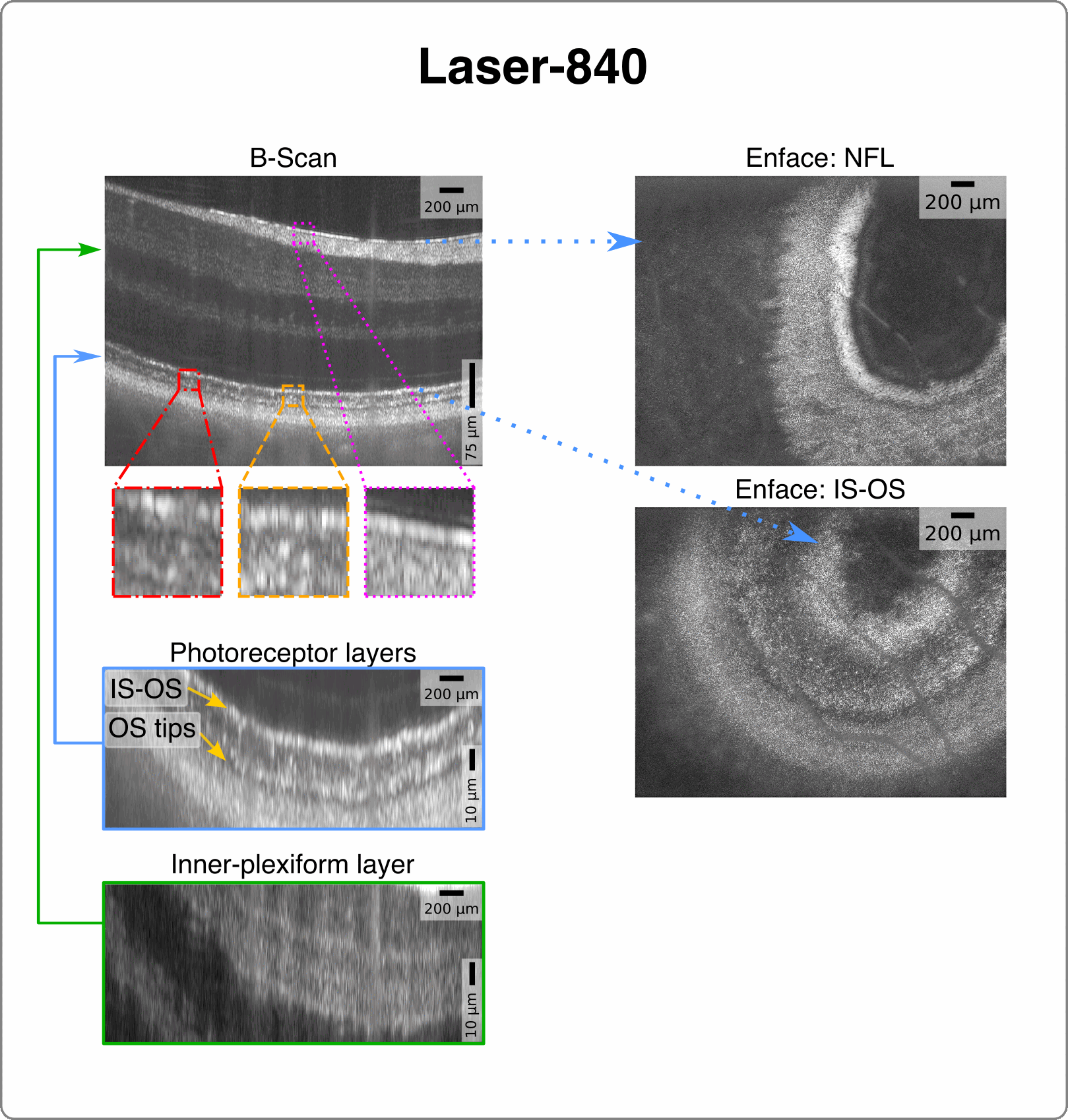

Supplement: Supplementary file 1 [file boe-17-2-686-v001.gif]
